# Morphology, biology and taxonomy of *Dendritobilharzia loossi* Skrjabin, 1924 (Trematoda: Bilharziellidae), a parasite of *Pelecanus onocrotalus* (Pelecanidae) and *Anas plathyrinchos* (Anatidae)

**DOI:** 10.1051/parasite/2011181039

**Published:** 2011-02-15

**Authors:** F.D. Akramova, D.A. Azimov, E.B. Shakarboev

**Affiliations:** 1 Laboratory of general parasitology, Institute of Zoology, Uzbek Academy of Sciences A. Niyazov 1 Tashkent 100095 Uzbekistan

**Keywords:** *Dendritobilharzia loossi*, trematode, life cycle, miracidium, cercaria, schistosomule, intermediate host, definitive host, *Dendritobilharzia loossi*, trématode, cycle biologique, miracidium, cercaire, schistosomule, hôte intermédiaire, hôte définitif

## Abstract

Life cycles of *Dendritobilharzia loossi* Skrjabin, 1924, a parasite of waterbirds, and its morphobiological traits are studied and described. Mollusks *Anisus spirorbis*, the infection rate of which in natural environments reaches 1.3-1.9%, were recorded as intermediate hosts under conditions of Uzbekistan. The development of this trematode in intermediate and definitive hosts lasts for 26 and 15 days, respectively. Diagnostic traits of the trematodes during all stages of their ontogeny are reviewed.

## Abstract (fr)

Le cycle biologique de *Dendritobilharzia loosi* Skrjabin, 1924, parasite d’oiseaux aquatiques, et ses caractéristiques morphobiologiques sont étudiés et décrits. Le taux d’infestation naturelle du mollusque hôte intermédiaire *Anisus spirorbis*, qui atteint 1,3 à 1,9 % dans l’environnement, a été analysé en Ouzbekistan. Le développement de ce trématode dans les hôtes intermédiaires et définitifs dure respectivement 26 et 15 jours. Les étapes de l’ontogenèse de ce parasite sont passées en revue.

## Introduction

The genus *Dendritobilharzia* Skrjabin et Zakharov, 1920 comprises trematodes parasitizing blood vessels of waterbirds. Currently, this genus includes four species, namely *Dendritobilharzia pulverulenta* (Braun, 1901), *D. loossi* Skrjabin, 1924, *D. anatinarum* Cheatum, 1941, and *D. asiatica* Mehra, 1940. Of these, the first two species were recorded in the Commonwealth of Independent States (the former USSR) including Uzbekistan (Skrjabin, 1951; Byhovskaya-Pavlovskaya, 1962; Iskova, 1968; Ryzhikov et al., 1974; Azimov, 1975; Smogorzhevskaya, 1976; Akramova & Azimov, 2005; and others). Until 1968, no data on the biology of the species from the genus *Dendritobilharzia* had been available in literature. It was not until 1968 that the first publications on the biology of one of the species from this genus, *D. pulverulenta* (Vusse, 1979, 1980) appeared in literature.

A detailed study of the morphology and biological traits of *D. pulverulenta* was carried out by (Khalifa, 1976). This author established the mollusks *Anisus vortex* (L., 1758) and *Planorbis planorbis* (L., 1758) as intermediate hosts of this trematode, the infection levels of which by the cercariae reached 5.6 and 1.7%, respectively. The mature parasites were recorded in the blood vessels of three nestlings of the domestic duck *Anas platyrhynchos* experimentally infected by *Dendritobilharzia pulverulenta*.

In the subsequent years, a significant interest was noted towards the study of the life cycles of trematodes from the genus *Dendritobilharzia*. Leite *et al*. (1982) studied the life cycles of *D. anatinarum*. The authors experimentally established the mollusks *Biomphalaria straminea* as intermediate hosts of this trematode. The cercariae left the mollusks 25 days post infection. The trematodes reached sexual maturity in blood vessels of domestic ducks 39 days post infection with cercariae.

The goal of the present work is the study of all stages of the development of morphological and biological traits of the trematode *D. loossi*.

## Material and Methods

**Fig. 1.**
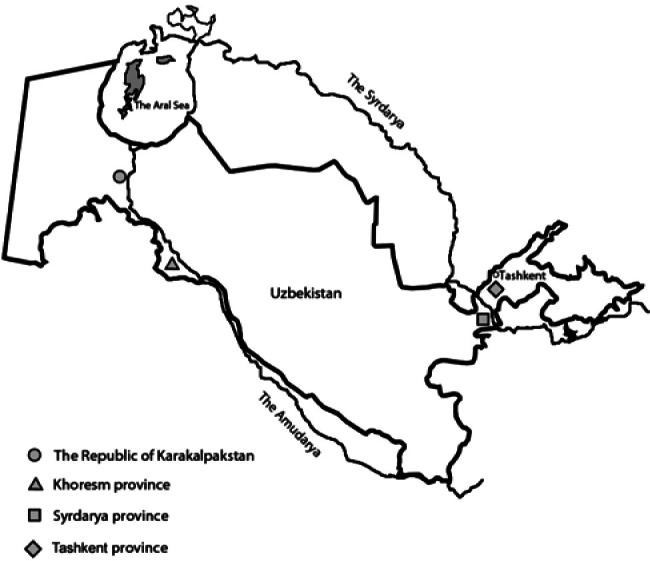
Map of Uzbekistan: locations of material collection.

The material of this study is comprised of our own collection of mollusks carried out in 2000-2007 from waterbodies situated in the floodplain of the Syrdarya River (in Tashkent and Syrdarya provinces) and in the lower reaches of the Amudarya River, where farms of Khoresm province and the Republic of Karakalpakstan are situated (see map, Fig. 1).

The collection of the mollusks was carried out according to a routine method described by Zhadin (1952) from the waterbodies of Gulistan, Bekabad and Chinaz districts, which are situated in the floodplain of the middle course of the Syrdarya River. Similar collections were carried out in the waterbodies of the lower reaches of the Amudarya River. Waterbodies situated in the floodplain were surveyed, namely, the system of Dautkul lakes, Lake Mashankul, Shegekul, Sudochie, Khodjakul and the system of Karadjar lakes. Totally, collected and studied were 5,600 freshwater mollusks, namely *Lymnaea auricularia*, *L. stagnalis*, *L. truncatula*, *Planorbis planorbis*, *Anisus septemgyratus*, *A. spirorbis*, *Physa fontinalis*, in spring, summer and autumn.

The parasite eggs isolated from naturally infected ducks *Anas platyrhynchos* dom., in the farm Saikhun situated in the Syrdarya province (25 July 2000), were used as the material for the reproduction of the biological cycle of development of the trematode *D. loossi*. We monitored the process of *D. loossi* miracidia at the following temperatures: 50, 40, 35, 30, 25, 20, 15, 10 and 5 °C. At the temperature 50 °C the eggs die quickly. Therefore, no hatching takes place. The same situation was recorded at the temperature of 40 °C.

Of seven studied ducks, which were dissected using the complete helminthological method of dissection as described by Skrjabin (1928), in one female we found 13 ♂ and 11 ♀ *D. loossi* in the vessels of the mesentery and liver. Besides, we found the eggs typical of *D. loossi* in the faeces.

The examination of the white pelican female (*Pelecanus onocrotalus*), which was shot by poachers on Lake Sudochie on 2 August 2002, revealed mature trematodes *D. loossi*, 5 ♂ and 3 ♀ in the vessels of the mesentery and liver.

The identification of the trematode *D. loossi* from ducks and a pelican was carried out by using the preparation stained using known helminthological methods. We studied 18 ♂, 14 ♀ of natural populations of adults from the ducks and the pelican and 30 ♂, 25 ♀ mature trematodes from experimentally infected birds.

For the infection of the mollusks we used the eggs of *D. loossi* obtained from naturally infected ducks *Anas platyrhynchos* dom. In laboratory conditions, miracidia emerging from the eggs were used for the artificial infection of fresh-water mollusks. The experimental infection of the mollusks by *D. loossi* miracidia was carried out on both individual specimens and groups. At the individual infection, each mollusk was put in a Petri dish into which one to three active miracidia were placed. One day later, 25-30 mollusks were transferred into each of small aquariums and monitored. During the group infection, the mollusks were kept in mid-sized aquariums (ca. 75-100 individuals per aquarium). Eggs containing mature miracidia were added to the aquariums.

Morphological and biological peculiarities of miracidia and cercariae were studied by using a generally accepted technique (Ginetsinskaya & Dobrovolsky, 1963; Ginetsinskaya, 1968). For the study of the morphology of miracidia (25 individuals) and cercariae (30 individuals) we used vital stains. Morphometric parameters of cercariae were studied on anesthetized solutions of the neutral red. While studying live objects we used vital stains: the neutral red, the sulphate of Nile blue, acetic carmine, toluidine blue, etc.

For the study of the morphology of live miracidia and cercariae, we used the phase-contrast microscopy. We used the method of silvering for the revealing of the borders between epithelial plates and sensitive papillae (sensilla) of miracidia, as well as of sensilla of cercariae. Miracidia were recorded at 0.5% or hot 1% nitro-acid silver, after which they are placed into glycerol and studied visually. The hatching of miracidia of the considered species from eggs takes place in the water (external environment), which depends on a number of abiotic factors.

Therefore, we studied the effect of various temperatures and illumination on the process of hatching of miracidia and emission of cercaria of *D. loossi*. We traced the emission of cercariae at the following temperatures: 45, 40, 35, 25, 20, 15, 10 and 5 °C during the natural alternation of the day and night. The experiments were conducted in three replications.

Material collected during 2000 to 2007 was used in the work. Experiments were repeated many times. The data given in the Tables I- IV and in the text (daily rhythms of cercariae emission) reflect average values.

**Table 1.** The effect of temperatures on the process of hatching of *D. loossi* miracidia from eggs

Temperature (in °C)	Number of eggs	Emergence of miracidia (%)	Time of emergence of miracidia (in minutes)
50	35	0	–
40	37	0	–
35	50	90	20–23
30	53	100.0	25–27
25	45	100.0	28–30
20	34	100.0	30–33
15	44	40.0	35–45
10	38	0	–
5	40	0	–

The birds were infected with cercariae that had emerged from mollusks. In the experiment, we used 20 nestlings of each of domestic ducks, geese and chickens at the age of 18-20 days, all of which were grown in conditions preventing their infection with the above mentioned infection. These birds were infected with cercariae (150-200 individuals per bird). The studies were carried out using modern equipment: the phasecontrast device, inverted CK2-TR (Olympus Japan), research microscope LOMO, cooling centrifuges TR7 (Dupont, USA), binocular ML-2200 (Olympus Japan).

## Results

### Eggs and miracidia

**Fig. 2.**
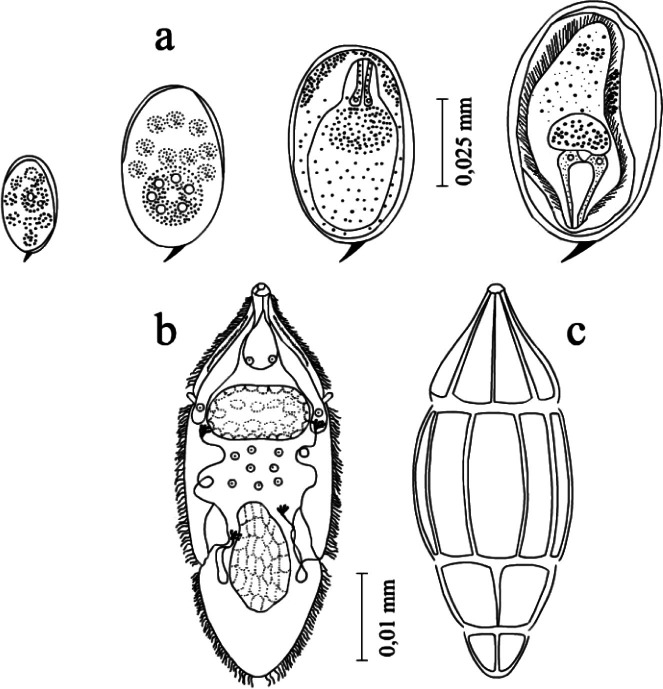
*Dendritobilharzia loossi* Skrjabin, 1924. a: consecutive stages of the embryo development; b: miracidium; c: the position of epithelial plates.

Trematodes lay eggs in the lumen of the capillaries in the intestine and other organs. Embryos develop in the eggs situated in the tissues of the definitive host (Fig. 2). Newly-shed eggs, oval with a sharp spine on one pole, are 0.066-0.074 × 0.02-0.03 mm. Mature eggs secreted with the excrements of birds are 0.10-0.12 × 0.04-0.06 mm; they are light-brown. Miracidia hatch during the contact of the egg with water. The optimal temperature providing the emergence of miracidia was 26-32 °C in our experiment. Shortly before the emergence from the egg a miracidium makes very active movements. In a bright solar light, most of miracidia emerge from the eggs in 25-45 min. At the temperature of 35 °C, miracidia hatch from 90% of eggs, while 10% of miracidia died inside the eggshell. The temperature of 25 °C to 32 °C is the most optimal for the hatching of miracidia (Table I). Lower temperatures (from 5 °C to 10 °C) are insufficient for the miracidia from the eggs. The hatching of active miracidia from the eggs was recorded at a gradual increase in temperatures to 25 °C and 32 °C.

It is noteworthy that illumination stimulates the process of emergence of miracidia from the eggs. A long-term maintenance of eggs with miracidia ready to hatch in the darkness results in their weakening and a significantly lower percentage of their hatching.

Our multiple observations that the hatching of miracidia takes place in a different way. It is known that the eggs of these trematodes have no cap. By the moment of the hatching of a larva, the egg as a rule splits in its anterior third, where the shell may be thinner than in the other parts and the miracidium moves out through the crack.

An actively moving miracidium of *Dendritobilharzia* has an elongated body slightly sharpened in the anterior and tapering in the posterior part. Miracidia show a positive photo- and negative geotaxis (Azimov, 1986). The time of the active life of larvae at the temperature of 26-32 °C is 18-20 hours.

The length of the miracidia reaches 0.09-0.10 mm at the maximal width 0.06 mm. The surface of the body is formed of four rows of epithelial plates, which have numerous cilia. The epithelial plates are situated by the formula 6:8:4:4 = 22. The part of the body free from cilia is relatively small, in which the duct of the apical gland opens up. A large apical gland is situated in the anterior part of body. A rather large cerebral ganglion of an elongated-oval shape is situated at the level of epithelial plates of the second row. Twelve sensillae lie on the border of the epithelial plates. There are two pairs of ciliary cells. The first pair lies along the sides of the cerebral ganglion; the second one in the posterior part of the miracidium. These cells are interconnected by convoluted tubules.

### Development in the organism of an intermediate host

**Fig. 3.**
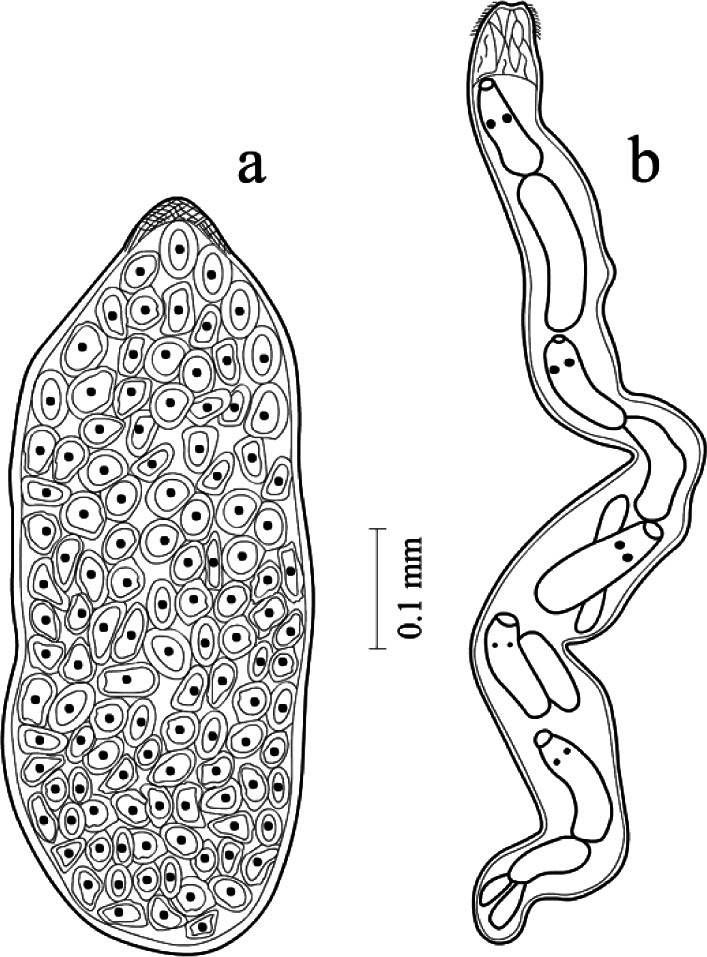
*Dendritobilharzia loossi* Skrjabin, 1924. Development of the trematodes in the intermediate host. a: mother sporocysts; b: daughter sporocysts.

The mollusk *Anisus spirorbis* (L., 1758) was recorded as the intermediate host of *D. loossi* both in the wild and in the experiment (Table II). Penetrating into the body of the intermediate host, miracidia of *Dendritobilharzia* undergo a regressive metamorphosis and turn into a mother sporocyst, an organism, which is characterized by an extremely simple structure. They breed parthenogenetically, giving rise to morphologically more complex individuals of the next generation – the daughter sporocysts (Fig. 3). The total infection rate of mollusks *A. spirorbis* by the *Dendritobilharzia* cercariae in natural conditions reached 1.3 and 1.9%. The cercariae were recorded in mollusks in summer (July-August) and in autumn (September). During the experimental infection, cercariae are formed in daughter sporocysts in the hepatopancreas of *A. spirorbis* mollusks.

**Table 2.** Infection of mollusks by cercariae of *Dendritobilharzia* under natural conditions.

		Studied individuals		
Mollusk species	Place of collection (province)	Numbers	Infected	Prevalence of invasion (%)
*Anisus*	Syrdarya	215	3	1.3
*spirorbis* (L)	Tashkent	105	2	1.9
*Planorbis*	Syrdarya	108	–	–
*Planorbis* (L)	Tashkent	110	–	–
*Lymnaea*	Syrdarya	126	–	–
*auricularia* (L)				

As the experiments showed, the time of the development of parthenitae and formation of cercariae in intermediate hosts depend on the temperature (Table III). So, at relatively high temperatures the formed cercariae began emerging from *A. spirorbis* in 26 days. The emission of cercariae continued until the death of infected mollusks.

**Table 3.** Effect of temperatures on time of emission of cercariae *D. loossi* from mollusks.

	Number of mollusks	Average daily air temperature (°C)	Infection rate (%)	Beginning of emergence of cercariae (days)
*A. spirorbis*	28	26–32	100	26
	31	22–26	100	29
	18	20–23	100	33
*P. planorbis*	30	26–32	100	27
	35	22–26	100	27
	27	20–23	100	29
*A. vortex*	35	26–32	100	26
	33	22–26	100	28
	38	20–23	100	32

### Cercaria

**Fig. 4.**
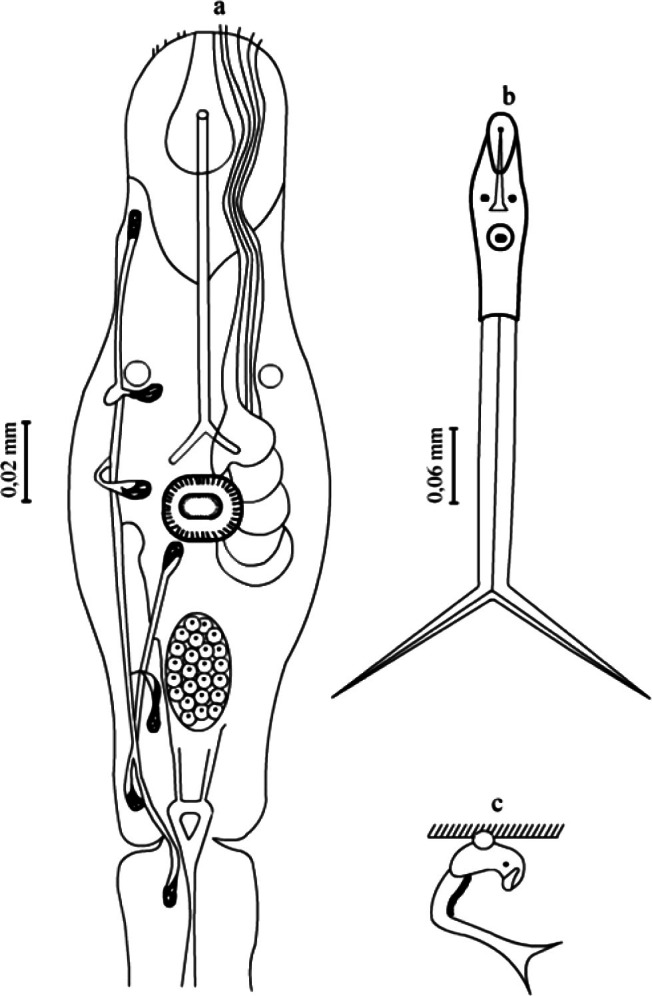
*Dendritobilharzia loossi* Skrjabin, 1924. a: details of the organs of cercaria; b: common view; c: resting posture.

The body of a cercaria is elongated-oval in shape, rounded on the anterior side (Fig. 4). The body length 0.220-0.246 mm; the width 0.063-0.078 mm. The anterior organ is elongated-oval in shape (the length 0.070-0.082 mm; the width 0.046-0.052 mm).

The ventral sucker is significantly shifted from the midpart of the body backwards. It is roundish in shape reaching 0.032-0.034 × 0.028-0.030 mm. The tail stem is 1.5 as long as the body and reaches 0.260-0.370 mm at the width of 0.026 mm. Tail furcae are shorter than the tail stem; their length is 0.126-0.146 mm. There is no natatorial membrane on the furcae (Table IV).

**Table 4.** Main morphometric differences in the cercariae *D. loossi* (sizes in mm).

	Our own data	Khalifa’s data (1976)	
Value	*A. spirorbis*	*P. planorbis*	*A. vortex*
Body length	0.220–0.246	0.232–0.243	0.174–0.220
Body width	0.063–0.078	0.069–0.081	0.050–0.063
Tail length	0.260–0.370	0.371	0.224–0.371
Tail width	0.026	0.023–0.029	0.023–0.034
Length of furca	0.126–0.146	0.127	0.127–0.150
Width of furca	0.010–0.016	0.011–0.017	0.017–0.029
Anterior organ	0.070–0.082 × 0.046–0.052	0.085–0.089 × 0.052–0.055	0.069–0.080 × 0.046–0.048
Ventral sucker	0.032–0.034 × 0.028–0.030	0.034 × 0.027	0.032 × 0.029

The digestive system consists of the oesophagus and two rudimental intestinal branches, which reach the first pair of the penetration glands. The latter are large, their number reaching five, of which two are preacetabular and three postacetabular pairs. There are two pigmented eyes. The excretory system is described in the formula 2[(1 + 1+1) + (3 + 1)] = 14. The excretory bladder is small.

**Fig. 5.**
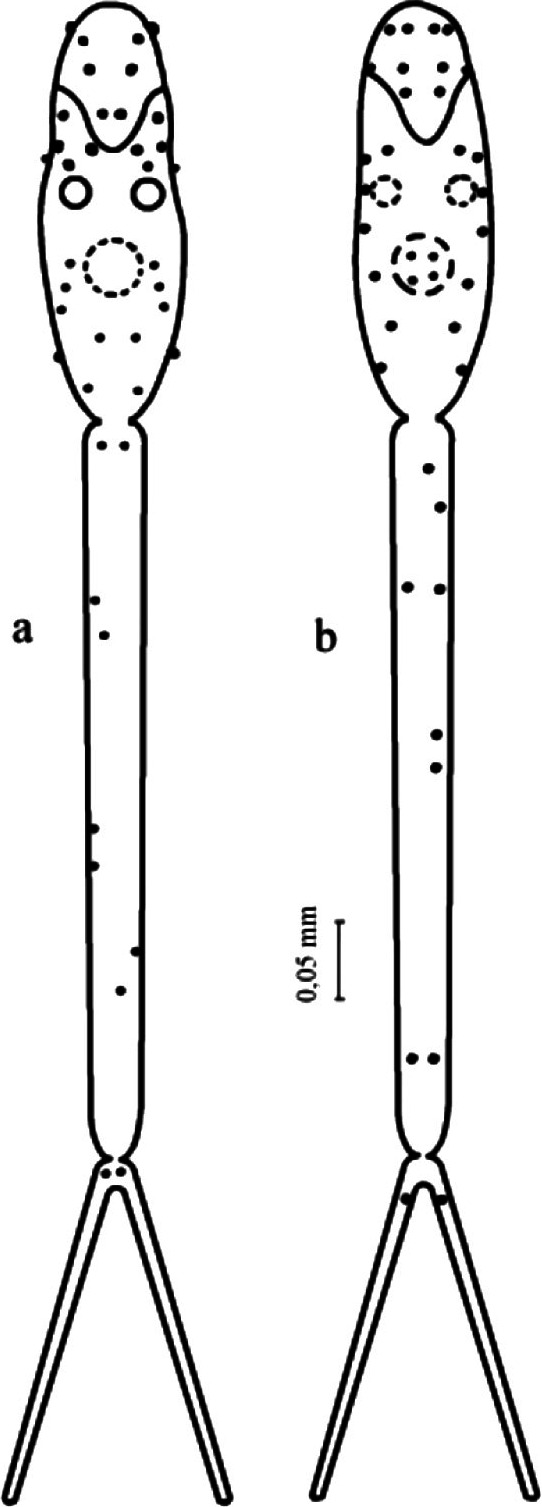
*Dendritobilharzia loossi* Skrjabin, 1924. Cercariae. a: dorsal-lateral sensillae; b:ventral sensillae.

The sensor apparatus consists of the dorsal, lateral and ventral complexes (Fig. 5). Sensillae are situated in symmetrical rows along the body, tail stem and furca. In general, the dorsal complex is formed of 34 sensillae; lateral, 6; ventral, 38, the total number of sensillae reaching 78.

Cercariae emerging from the mollusks into the water are very active. The cercariae *D. loossi* has the ability of moving both the anterior end and posterior forward. This movement is the result of the contraction of the musculature of the tail stem. The furcae of the tail play the role of a rudder.

Cercariae show a positive photo- and negative geotaxis (Azimov, 1986). The most intensive emission from the mollusks takes place in the morning and in the afternoon. An increase in illumination and temperature contributes to an intensive emission of cercariae from the intermediate host.

The studies have shown that the emission of cercariae from the host mollusk is stimulated or hindered by the effect of environmental factors, of which the most important are the temperature and light. Inter-relations of these two factors provide the periodicity of the emission of cercariae. As the results of the study shows, the emission of the cercariae from mollusks takes places at a certain temperature, on average from 15 to 35 °C. The death of host mollusks is recorded at high temperatures (40-45 °C). At 15 °C the emission of cercariae is significantly restricted, while at 10-5 °C the emission of the cercariae ceases. The optimal temperature for the emission of cercariae from host mollusks is 25-32 °C.

The pattern of the curve of the daily rhythm of cercariae emission has two peaks. The first maximal peak is noted at 2 pm, while the second at 7 pm. Thus, the intensity of the emission of cercariae depending on the light and temperature to a certain degree connected with the pattern of their taxis. The cercariae of this species are characterized with a positive photo- and thermotaxis.

The cercariae emitted from the mollusk produce quick movements. The resting posture is very characteristic. They hang motionless, attached with the sucker to the film of the water surface tension. At this point, the ventral sucker becomes elongated and the body bends dorsally, while the tail stem and furcae hang. When soaring, the furcae are significantly straightened out. The duration of the cercariae in the water is about two days.

### Development of cercariae in definitive hosts

A series of experiments on the experimental infection of birds with cercariae *D. loossi* in laboratory conditions was carried out, which was aimed at obtaining of the original material for the reproduction of the complete life cycle of *Dendritobilharzia* in laboratory conditions.

Experiments on the infection of goslings and chickens yielded a negative result. Nineteen ducklings became infected. On days 18 and 20 mature eggs of *Dendritobilharzia* were recorded in their faces. After this experiment we started exploring the ontogeny of this trematode in the definitive host (Table V).

**Table 5.** Time of development of *D. loossi* in the definitive host (experiment).

Number of ducklings in experiment	Number of cercariae in one bird	Found individuals		Time of autopsy desiccation from the beginning of experiment (days)
		♂	♀	
		Schistosomules		3
4	150-200	Schistosomules		4
		Schistosomules		5
		Schistosomules		10

		53	–	12
3	150–200	–	44	13
		–	52	14

		37	23	15
3	150–200	–	49	25
		41	18	60

**Fig. 6.**
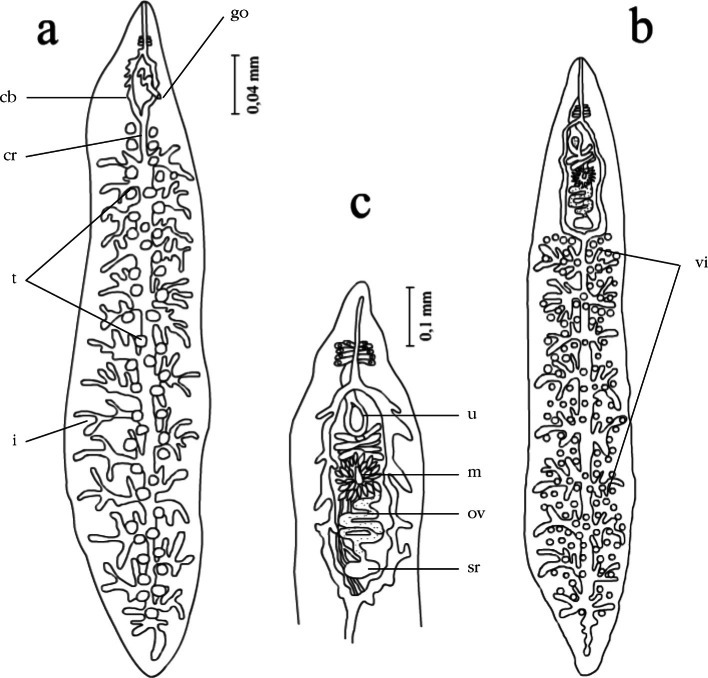
*Dendritobilharzia loossi* Skrjabin, 1924. a: male; b: female; c: topography of sexual organs in females. Abbreviations. cb: caeca bifurcation; cr: caecal reunion; t: testes; i: intestine; u: uterus; m: Mehlis’ gland; ov: ovary; sr: seminal receptacle; vi: vitelline.

The study of birds revealed schistosomules in blood vessels of the lungs 72 hours post infection, and in the liver and kidney 5 and 10 days post infection. It was established that the differentiation of trematodes into males and females was manifested on day 12 post infection. On day 15 the trematodes reached sexual maturity, when males and females had completely formed sexual organs (Fig. 6). Mature males and females were found in the blood vessels of the mesentery, kidney, liver and intestine as a result of the study of ducks by the method of the complete helminthological autopsy. Eggs at varying stages of development were recorded under the intestinal mucus.

Thus, we for the first time described the life cycle of the trematode *D. loossi*. The circulation of the infection in biocenoses takes places according to the following scheme: adult → egg → miracidium → intermediate host → cercaria → definitive host.

## Discussion

The female *D. loossi* was described by Skrjabin (1924) from blood vessels of *Pelecanus onocrotalus* in Kazakhstan. In 2000, we found this species in *Anas platyrhynchos* dom. from the waterbody of Syrdarya province in the Republic of Uzbekistan and in *P. onocrotalus* from Lake Sudochie in the Republic of Karakalpakstan (Akramova & Azimov, 2005). There is no additional information on the records of *D. loossi*. This convinces us of the restricted distribution of *D. loossi*, the ranges of the population of this species covering the territory of Kazakhstan and Uzbekistan. The growing interest to this group of trematodes in the last few years enabled a deeper study of the biology of *Dendritobilharzia*, particularly their life cycles and morphology at all stages of development. In a relatively short period, researchers from different states have almost completely studied the life cycles of a number of species, *e.g. D. pulverulenta* (Khalifa, 1976; Vusse, 1979, 1980), *D. anatinarum* (Leite *et al*., 1982), *D. loossi* (our data). The results of these studies have expanded our knowledge of this group of trematodes and helped to reveal the most significant specific peculiarities of the biology of each of indicated species. In this connection, of certain interest are materials on the morphology and biology of *D. loossi*.

The entire course of *D. loossi* biology passes according to the known scheme typical of the genus *Dendritobilharzia*.

The adults of *D. loossi* are obligatory parasites of pelicans *P. onocrotalus* and ducks *A. platyrhynchos* dom. Trematodes are localized in the lumen of blood vessels of the intestine, liver and kidneys. The trematodes reach sexual maturity on 18-20^th^ day post infection. Mollusks *Anisus spirorbis* were recorded as intermediate hosts under natural conditions of Uzbekistan.

Miracidia *D. loossi* actively penetrate into mollusks, intermediate hosts. The development and maturation of cercariae in sporocysts depend on the temperature and last 26-33 days. The emission of cercariae is regulated by different environmental factors. The intensity of the emission directly depends on the temperature and illumination. As the results of the original studies of *D. loossi* and literature on the biology of *D. pulverulenta* and *D. anatinarum* showed, parthenogenetic generations of these trematodes are characterized with a strict specificity towards their host. So, larval stages of *D. loossi* develop only in the mollusk *A. spirorbis*; *D. anatinarum* are confined only to mollusks *B. straminea*, while *D. pulverulenta* to mollusks *P. planorbis* and *A. vortex*.

The rate of the development of the larval stages depends on the habitat of the mollusk host and factors of external environments. The most important is the factor of temperature. The studies of *D. loossi* showed that an increase in the temperature accelerates, while a decrease in it slows down the development of parthenitae and formation of cercariae. These conditions and interaction of taxis enables the concentration of cercariae in certain zones of a waterbody. At contact with definitive hosts, a cercaria actively penetrates into blood vessels through their cover.

The analysis of morpho-biological peculiarities of *D. loossi* at all phases of ontogeny shows that a number of traits in the process of development do not undergo significant changes; in males, the length and configuration of the gynaecophoric canal, the number and position of testes; in females, the form of the ovary and uterus; the number of eggs in the uterus; the form and ornamentation of eggs. The following traits of cercariae can serve as species criteria: the number and position of ciliary cells and penetration glands.

The results of conducted studies on morpho-biological traits of *D. loossi* enabled the consideration of some disputable points of the taxonomy of individual species of the genus *Dendritobilharzia*.

The genus *Dendritobilharzia* comprises four species, namely *D. pulverulenta*, *D. anatinarum*, *D. asiatica* and *D. loossi*, which parasitize blood vessels of birds. The definitive hosts are birds of the orders Pelecaniformes, Anseriformes and Gruiformes. Mollusk species from the family Planorbidae were recorded as their intermediate hosts.

Of the indicated species, the populations of *D. pulverulenta* are most widespread in Anseriformes and Gruiformes (Braun, 1901; Skrjabin, 1951; Bykhovskaya- Pavlovskaya, 1962; Ryzhikov et al., 1974; Palm, 1965; Ulmer & van de Vusse, 1970; Sulgostowska, 1972; Khalifa, 1976; Azimov, 1975; Kolarova et al., 1989, 1997; Rind, 1987; Akramova & Azimov, 2005; Bayssade- Dufour et al., 2006). The life cycle of this species under conditions of Poland, as Khalifa (1976) notes, passes with the participation of mollusks *Anisus vortex* and *Planorbis planorbis*. Morpho-biological traits of cercariae and maritae *D. pulverulenta* are characterized with a rather distinct difference from other species.

Of special attention are the results of recent studies carried out by Bayssade-Dufour *et al*. (2006), who established the range of the intraspecific variability of some traits in *D. pulverulenta*, depending on the season, hosts and geographic conditions. The morphology of recorded *D. pulverulenta* males and females from *Cygnus olor* in France significantly differed from the described specimens of this species from other geographic zones. Based on molecular studies the authors reasonably draw a conclusion about the identity of different forms and their belonging to the species *D. pulverulenta*.

The ranges of species *D. anatinarum* and *D. asiatica* cover specific territories in America and Asia, in which a limited number of Anseriformes species are registered (Cheatum, 1941; Freitas & Costa, 1972; Chauhan *et al*., 1974; Azimov, 1975; Leite *et al*., 1982; Borgarenko, 1984). The life cycle of *D. anatinarum* was studied by Leite *et al*. (1982) in Brazil. The authors established mollusks *Biomphalaria straminea* as intermediate hosts of this species. Thus, the results of the study of life cycles in *D. pulverulenta*, *D. anatinarum* and *D. loossi* show the specificity of the considered species to specific mollusk and avian species (Table VI). The specificity to hosts, in particular to mollusks, may serve as one of the arguments for the independence of the indicated species.

**Table 6.** A comparative characteristic of the morphology of cercariae *D. loossi*, *D. anatinarum* and *D. pulverulenta* (sizes in mm).

Trait – Cercaria	*D. loossi* (our data)	*D. anatinarum* (by Leite *et al.*, 1982)	*D. pulverulenta* (by Khalifa, 1976)
Body length	0.220–0.246	0.169–0.207	0.174–0.243
Body width	0.063–0.078	0.09–0.016	–
Tail length	0.260–0.370	0.180–0.219	0.225–0.371
Furca length	0.126–0.146	0.53–0.74	0.118–0.266
Anterior organ	0.070–0.082 × 0.046–0.052	0.058–0.080 × 0.037–0.049	0.085–0.089 × 0.052–0.055
Ventral sucker	0.032–0.034 × 0.028–0.030	0.019–0.027 × 0.021–0.030	0.034 × 0.027
Cuticular arming	available	available	available
Formula of excretory system	2[(1 + 1+1) + (3 + 1)] = 14	2[(1 + 1+1) + (2 + 1)] = 12	2[(1 + 1+1) + (3 + 1)] = 14
N of penetration glands	5 pairs	5 pairs	5 pairs
Eyes	2	2	2
Intermediate hosts (Planorbidae)	*Anisus spirorbis*	*Biomphalaria straminea*	*Planorbis planorbis Anisus spirorbis*

It is noteworthy that Macko (1959) and Vusse (1980) consider *D. anatinarum* as a synonym of *D. pulverulenta*. Azimov (1975) does not share this viewpoint of Macko and Vusse and recognizes the independence of the species *D. anatinarum* and *D. pulverulenta*, which are quite easily differentiated one from another by the structure of the uterus and ovaries. Agreeing with the views of Skrjabin (1951) and Farley (1971) we recognize the validity of species *D. pulverulenta* and *D. anatinarum*. The results of recent experimental studies of the morphology and biology of *D. anatinarum* by Leite *et al*. (1982) from Brazil support this conclusion. These authors established the mollusks *B. straminea* as intermediate hosts of this species and domestic ducks *Cairina moshata* dom. as definitive hosts. By a number of traits of mature forms and cercariae *D. anatinarum* and *D. pulverulenta* are clearly differentiated.

Unfortunately, the authors (Macko, 1959; Vusse, 1979, 1980) did not justify their viewpoint properly. In their conclusions they were based on insignificant differences in the traits of maritae *D. anatinarum* and *D. pulverulenta*.

After the work by Khalifa, (1976) and Leite *et al*. (1982), who studied morpho-biological peculiarities of the indicated trematodes, the erroneous views of Macko (1959) and Vusse (1979, 1980) became evident. They did not pay sufficient attention to the fact that the seminal vesicle in *D. anatinarum* males is elongated, while in *D. pulverulenta* it is strombuliform. Similar differences are present in the females in the structure of the uterus, ovary and egg form (Table VII). The most significant differences are noted in a number of traits in cercariae of these species (Table VI). The conservatism of the structure of the excretory system should be noted. Thus, the formula of this system are expressed in *D. anatinarum* as 2[(1 + 1+1) + (2 + 1)] = 12, while in *D. pulverulenta* as 2[(1 + 1+1) + (3 + 1)] = 14 (Khalifa, 1976; Leite *et al*., 1982). Therefore, we cannot agree with the opinion of Macko and Vusse on the synonymy of *D. anatinarum* and *D. pulverulenta*, and consider the former species as the independent one.

**Table 7.** – A comparative characteristic of the morphology of *D. loossi*, *D. anatinarum*, *D. pulverulenta* and *D. asiatica* (sizes in mm).

	Trait	*D. loossi* (our data)	*D. anatinarum* (by Leite *et al*., 1982)	*D. pulverulenta* (by Khalifa, 1976)	*D. asiatica* (by Chauhan *et al*., 1973)
♂	Body length	9.5–12.5	6.2	3.25–8.83	Male not described
	Body width	1.35–1.86	0.60–0.75	0.40–1.79	
	N of testicles	138–148	120–130	90–110	
	Seminal vesicle	Strombuliform	Bulbus-like	Strombuliform	
♀	Body length	10.5–13.6	8.0	3.35–10.3	6.0
	Body width	1.32–1.38	0.89–1.01	0.45–1.65	1.3
	Ovary	Strombuliform	Elongated, tubular	Strombuliform	Long, convolute
	Uterus	Tubiform	Long with tortuous knots	Compact, roundish	Large, coiled

Number of eggs in uterus		One	Numerous	Numerous	Numerous

Shape of eggs		Oval with on spine on one pole	Roundish, without spines	Oval with spine on one pole	Oval with spine on one pole

Definitive hosts		*Anas platyrhynchos* dom.	*Anas platyrhynchos*	*Querquedula querquedula*	*Anas crecca*
		*Pelecanus onocrotalus*	*Cairina moschata* dom.	*Anas boschas*	
				*Fulica atra*	
				*Anas platyrhynchos*	

In general, morpho-biological traits of *D. anatinarum*, *D. pulverulenta* and *D. loossi* enable us to outline the main traits suitable for the identification of species of the genus *Dendritobilharzia* (Tables VI and VII). Note that morphometric traits turned out to be unfit for distinguishing these species. The ranges of their variability in all compared species (both males and females) are intersecting.

The considered species can be differentiated by the number of testes in males and the structure of the ovary, uterus and eggs in females. The most significant groups of traits of cercariae are the formulas of the excretory system, the number and pattern of the disposition of the penetration glands.

There are also differences in the biology of cercariae, which develop as the following: *D. loossi* in the intermediate host mollusk *A. spirorbis*; *D. anatinarum* in *B. straminea*; *D. pulverulenta* in *P. planorbis* and *A. spirorbis*. A strict specificity of *Dendritobilharzia* to their mollusk hosts can be confidently stated. Certain specificity was also noted for the adults of the considered species. So, the range of definitive hosts of *D. loossi* is confined to two species from the family Pelecanidae and Anatidae. The definitive hosts of *D. anatinarum* are two species of the genera *Anas* and *Cairina* (Anatidae). The range of hosts of *D. pulverulenta* is wider and includes the genera *Anas*, *Aythia*, *Fulica* and *Cygnus*. Only *Nettium crecca cressa* (= *Anas crecca*) was recorded as the definitive host of *D. asiatica*.

All the above mentioned materials suggest the peculiarity of the morpho-biological and ecological peculiarities of trematodes of the genus *Dendritobilharzia* at all phases of ontogeny. Summarizing the results of the conducted analysis it is possible to draw the following conclusions:

1. The taxonomical value of the entire complex of morphological traits in the adults and cercariae is not the same. By the diversity and taxonomic value of morphological traits more accent should be placed on the diagnostic criteria of cercariae. If studied in detail of their morphology, the identification of species does not pose special difficulties. To that end, it is necessary to use the single methodology of collection and processing of the material providing a high stability of morphological traits of cercariae.
2. In adults (males and females), the taxonomic value of traits is low due to a wide individual variability, which are connected with different factors.
